# Spontaneous Perforation of the Colon and Hypothyroidism: Report of a Case and Review of Literature

**DOI:** 10.4021/gr2010.06.212w

**Published:** 2010-05-20

**Authors:** Sanoop K. Zachariah, Nirmalan Raja

**Affiliations:** a Department of General, Gastrointestinal and Laparoscopic Surgery, M.O.S.C Medical College, Kolenchery, Cochin, Kerala, 682311, India

**Keywords:** Spontaneous perforation, Colorectal, Hypothyroidism, Hartmann’s procedure, Intestinal motility

## Abstract

Spontaneous perforation of the colon is a well documented but rare clinical entity commonly found to occur in the elderly and associated with chronic constipation. Hypothyroidism is known to be associated with intestinal motility disorders ranging from chronic constipation to more serious conditions like mega colon and pseudo obstruction. The case described here is that of a 35 years old hypothyroid male who presented with perforation peritonitis due to spontaneous perforation of the rectosigmoid. A thorough search of literature shows only one report of spontaneous perforation of the colon associated with hypothyroidism, to date. This case gives an opportunity to review the literature and discuss such unusual and dangerous presentations of hypothyroidism associated colonic motility disorders.

## Introduction

Spontaneous perforation of the colon (SPC) is a rare condition that occurs in the absence primary bowel pathology such as tumours, diverticulosis, chronic inflammatory disease or trauma [1]. This disease is more frequent in the elderly. The mean age at onset is more than 60. The mortality rate of this disease is as high as 35% to 47% [2, 3]. A thorough search of literature showed that an article in Hebrew, by Sirik Z. et al [4] to be the first and perhaps the only report to date, highlighting the association between SPC and hypothyroidism. The present case is probably the first report in English literature to document spontaneous recto-sigmoid perforation in a young hypothyroid patient.

## Case Report

**Figure 1:**
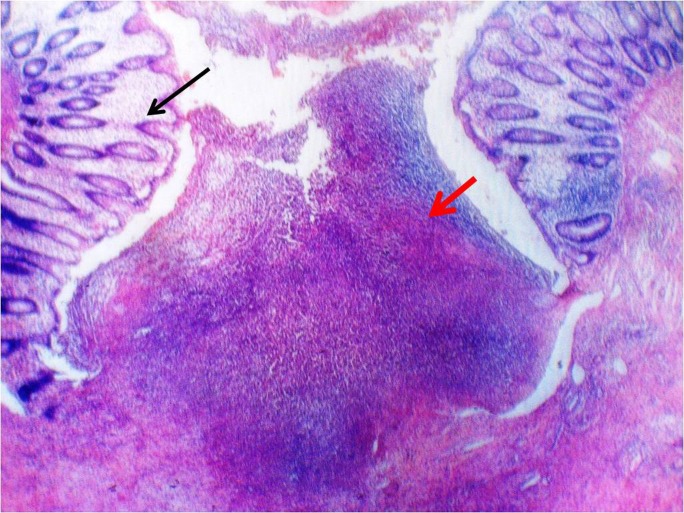
Photomicrograph showing the site of focal perforation with normal colonic tissue (black arrow), and ulcerated mucosa with inflammatory changes and debris (red arrow). (Haematoxylin and eosin staining, magnification x 4).

A 35 years old male, a known case of hypothyroidism for the past 3 years presented to the emergency department with upper abdominal pain of 16 hours duration. He was on thyroid hormone therapy for 2 years, which he discontinued and switched over to Ayurvedic (alternative medicine) treatment since one year. He gave a history of chronic constipation which worsened since the last four months. He was also on treatment for acid peptic disease. On examination he was tachycardic (pulse rate: 100/min), blood pressure was 110/70 mm Hg. The abdomen was distended with maximal guarding and tenderness in the epigastrium. Bowel sounds were diminished. Laboratory investigations showed a total count of 14,900/mm^3^. Thyroid hormone profile was T3 = 55 ng/dl (normal range 97 - 169), T4 = 3.5 ugm% (normal range 5.5 - 11), TSH = 22µ IU/ml (normal range 0.462 - 4.68). Plain abdominal X-ray revealed free intraperitoneal air under the right hemi diaphragm. Hence based on a provisional diagnosis of hollow viscus perforation, an emergency exploratory laparotomy was performed. At laparotomy the upper abdomen appeared relatively normal; however there was purulent fluid and multiple small fecoliths in the pelvis. A thorough search revealed a small circular perforation of about 1 cm diameter over the antimesenteric border of recto-sigmoid. The sigmoid colon was found to be thinned out and dilated with hard faecal matter within it. No other gross abnormality could be detected. The mesenteric artery pulsations were well felt. Hence a Hartmann’s procedure was performed using a linear stapling device, and a segment of about 10 cm of thinned out distal colon including the ulcer was resected. The post operative period was uneventful. Manual faecal evacuation and multiple enemas were given through the colostomy to evacuate impacted faeces. He was started on thyroid hormones 200 ug/day. Histopathology report was suggestive of a non specific ulcer with focal mucosal and sub mucosal neutrophilic infiltration (Fig. 1).

## Discussion

In 1827, Brodie described cases of middle-aged women whose rectums spontaneously ruptured [5]. In 1919 Huntley reported a case, with chronic constipation, which collapsed from a tear in the pelvic colon during defecation [6]. Only about 70 cases of SPC have been reported to date [7].

The exact aetiology of SPC is unclear. Stercoral ulcers of the colon, cortisone therapy, hypothyroidism, rectal prolapse, and psychiatric disorders have been suggested as contributory factors [1]. SPC can be classified into stercoral and idiopathic perforations [2]. Stercoral perforations are seen in patients with chronic constipation. The stagnant solid faecal mass compresses the colonic wall, and leads to ischemic necrosis of colonic mucosa, which forms an ulcer. Increased intraluminal pressure due to resistance in the rectum, together with voluntary contraction of abdominal musculature at defecation, leads to colonic rupture at this already weakened site. Idiopathic perforation occurs due to asymmetrical distribution of intraluminal pressure at the pelvirectal angle, in the absence of obvious impacted faecal matter.

SPC most frequently occurs at antimesenteric border of the rectosigmoid, which is an area of physiological ischemia. The lower water content of stool and also narrow intraluminal diameter are contributory factors. Maurer et al [8] reported that 52 out of 81 cases (64%) of feculent perforation occurred at these sites.

Hypothyroidism is well associated with intestinal hypomotility, chronic constipation, faecal impaction, atony, distension, and pseudo-obstruction [9]. Reduction of peristalsis is the main pathophysiologic process. Up to 15% of patients have fewer than 3 bowel movements weekly [10]. Goto S. et al [11] demonstrated colonic hypomotilty and dilated colon in hypothyroid rats. Shafer et al [12] demonstrated that gastrointestinal transit time improved significantly with thyroid replacement. Hypothyroidism may influence transepithelial flux transport by inhibiting CL^-^/HCO^3-^ anion exchange with a subsequent effect on intestinal motility [13].

In this case it could be assumed that hypothyroidism was the cause of chronic constipation which in turn led to faecal impaction. The hard stools would have caused a solitary pressure ulcer which later perforated spontaneously. Furthermore, SPC commonly occurs in the elderly, unlike in this case where hypothyroidism could be the most likely cause for occurrence at a young age. There was no other obvious pathology such as a tumour, diverticulitis or chronic inflammatory disease, in this case. Histopathological findings are usually non-specific, consisting of acute inflammatory reaction surrounding the perforation site [3]. In the present case the perforated area was surrounded by mild inflammatory infiltrate of polymorphonuclear neutrophils in the mucosa, while the adjacent colonic wall appeared normal, thereby ruling out inflammatory bowel disease. There was no evidence of tuberculosis. Ischemic colitis was ruled out as the mesocolon revealed normal blood vessels. The diagnosis of spontaneous perforation is based on the exclusion of primary bowel pathology and external injury including iatrogenic injuries.

The treatment for spontaneous perforation of the rectosigmoid is based on the same principles as other perforations of the rectosigmoid. Surgical options include primary closure, with or without proximal diverting colostomy and Hartmann surgery. In this case a Hartmann’s procedure was performed as a segment of relatively thinned wall unprepared faecal loaded colon had to be removed. Serpell et al [2] found that the complication rates after Hartmann surgery was lower than in case of other operations.

In conclusion, SPC is a rare but noteworthy condition. It can be rationally assumed that SPC is a dangerous complication of the colonic motility disorders associated with hypothyroidism. Surgical options are same as for other causes of rectosigmoid perforations. A high index of suspicion is needed to correctly diagnose this condition. Symptoms of gastrointestinal dyskinesia due to hypothyroidism should not be taken unconscientiously.
